# Mechanical and in vitro biological properties of uniform and graded Cobalt‐chrome lattice structures in orthopedic implants

**DOI:** 10.1002/jbm.b.34857

**Published:** 2021-05-08

**Authors:** Stefania Pagani, Erica Liverani, Gianluca Giavaresi, Angela De Luca, Claudio Belvedere, Alessandro Fortunato, Alberto Leardini, Milena Fini, Luca Tomesani, Paolo Caravaggi

**Affiliations:** ^1^ Complex Structure of Surgical Sciences and Technologies IRCCS Istituto Ortopedico Rizzoli Bologna Italy; ^2^ Department of Industrial Engineering Università di Bologna Bologna Italy; ^3^ Movement Analysis Laboratory IRCCS Istituto Ortopedico Rizzoli Bologna Italy

**Keywords:** additive manufacturing, biocompatibility, graded lattice structures, orthopedic implants, osteoblasts

## Abstract

Human bones are biological examples of functionally graded lattice capable to withstand large in vivo loading and allowing optimal stress distribution. Disruption of bone integrity may require biocompatible implants capable to restore the original bone structure and properties. This study aimed at comparing mechanical properties and biological behavior in vitro of uniform (POR‐FIX) and graded (POR‐VAR) Cobalt‐chrome alloy lattice structures manufactured via Selective Laser Melting. In compression, the POR‐VAR equivalent maximum stress was about 2.5 times lower than that of the POR‐FIX. According to the DIC analysis, the graded lattice structures showed a stratified deformation associated to unit cells variation. At each timepoint, osteoblast cells were observed to colonize the surface and the first layer of both scaffolds. Cell activity was always significantly higher in the POR‐VAR (*p* < 0.0005). In terms of gene expression, the *OPG/RANKL* ratio increased significantly over time (*p* < 0.0005) whereas *IL1β* and *COX2* significantly decreased (7 day vs 1 day; *p* < 0.0005) in both scaffolds. Both uniform‐ and graded‐porosity scaffolds provided a suitable environment for osteoblasts colonization and proliferation, but graded structures seem to represent a better solution to improve stress distribution between implant and bone of orthopedic implants.

## 1 INTRODUCTION

Human bones can be classified as functional lattice graded materials with the external cortical layer providing the bone with the overall mechanical properties and the sufficient strength to withstand mechanical loading,^1^, ^2^, ^3^, ^4^ and the internal porous trabecular structure allowing for even stress distribution across bone epiphysis^5^, ^6^, ^7^, ^8^ and hosting hematopoietic bone marrow and vascularization of the tissue.^9^ Disruption of bone integrity and morphology due to traumatic events, bone defects, removal of tumors and, at the epiphysis of long bones, to severe joint osteoarthritis, may require biocompatible implants such as osteosynthesis and fixation devices and/or endoprostheses capable to restore the original bone structure and its mechanical properties.^10^ Load bearing implants, such as joint endoprostheses, are particularly critical for the mechanical loading that these must sustain whilst preserving the physiological range of motion and the multiplanar mobility of the intact joint. In the lower limb, these implants must withstand large‐magnitude dynamic loadings up to four times the body weight according to the motor task,^11^ and must be wear‐resistant in a biological environment. In case of joint replacement, the need for strong primary fixation of the implant with maximum preservation of the original bone stock, and for minimization of the stress shielding due to the different mechanical properties with respect to those of the bone,^12^, ^13^ has pushed the research for lattice graded materials with appropriate mechanical and osteointegration properties.^14^, ^15^, ^16^, ^17^, ^18^

For orthopedic implant applications, uniform or graded porosity scaffolds can be obtained from the repetition of unit cells with different geometrical shapes and density as to mimic the radially graded porosity of the human long bones.^19^ Due to its good mechanical properties and elasticity, titanium alloys have long been used for most orthopedic implants and fixation devices, with the exclusion of the load‐bearing implants used for joint replacement for which Cobalt‐chrome alloy (CoCr) alloys are generally the materials of choice. The high modulus of elasticity of CoCr, around twice as large as that of titanium alloys, is a key property to provide implants with sufficient strength to bear physiological loadings but may result disadvantageous in terms of stress shielding. Therefore, design optimization of the implant‐to‐bone interface of CoCr endoprostheses is particularly critical. In addition to providing implants with the proper mechanical behavior, a porous interface allows for primary and long‐term fixation to the hosting bone thus a good osteointegration should be guaranteed. Pores size, overall porosity and interconnectivity are critical properties affecting cells migration within the implant, promoting the growth and avoiding overcrowding, allowing the passage of nutrients and of oxygen supply, and removing metabolic waste.^10^ While the optimal pore size of structures interacting with some biological tissues has been identified, the optimal porosity of the implant‐to‐bone interface of orthopedic implants is still controversial.^20^, ^21^, ^22^ Nevertheless, it is now generally accepted that lattices with pore diameters between 300–1,000 μm provide bone cells with suitable environment for viability and proliferation, regardless of the unit cells type.^23^ It has been observed that structures with pore size larger than 300 μm are advantageous in terms of cell proliferation and deep colonization, and these beneficial effects override the initial better cell attachment induced by smaller pores.^24^

Despite the extensive literature on this topic, covering the optimal porosity^25^, ^26^ and the fatigue behavior of lattice structures also via topological modelling,^27^, ^28^ it is still unclear whether biomimetic graded scaffolds are advantageous with respect to uniform density scaffolds in terms of osteointegration and minimization of the stress shielding between implant and bone. Functionally graded lattice structures obtained by the repetition of unit cells of varying sizes and shapes according to the local functional request of the implant^29^ should be exploited to improve osseointegration and to limit stress shielding failures.^30^, ^31^, ^32^ Low density structures have already been shown to be apt for bone cells proliferation and, in terms of mechanical interaction, may help orthopedic implant and prosthesis components to better conform with the overall bone stiffness. A gradual increase of volumetric density, from the inner region of the implant to the external surface of the endoprosthesis, is a feasible design solution to adjust the mechanical properties promoting correct load and stress distribution between implant and bone.

While the effect of unit type and porosity on the mechanical properties and interaction with biological tissues in vitro and in vivo has been largely investigated for Titanium alloys scaffolds,^25^, ^33^, ^34^, ^35^, ^36^ the current knowledge on mechanical and biological properties of functionally graded CoCr lattices is still limited.^19^, ^37^ This study aimed at providing novel information on the mechanical and biological behavior of CoCr lattice that may be used as material for orthopedic implants. Moreover, we aimed at identifying possible differences between uniform‐ and variable‐porosity scaffolds presenting the same material, unit cell and average porosity. The latter may be used to gradually decrease the stiffness of the implant interface closer to the bone, thus helping to decrease the stress shielding of endoprostheses.

## 2 MATERIALS AND METHODS

### 2.1 Design and manufacturing of the samples

**FIGURE 1:**
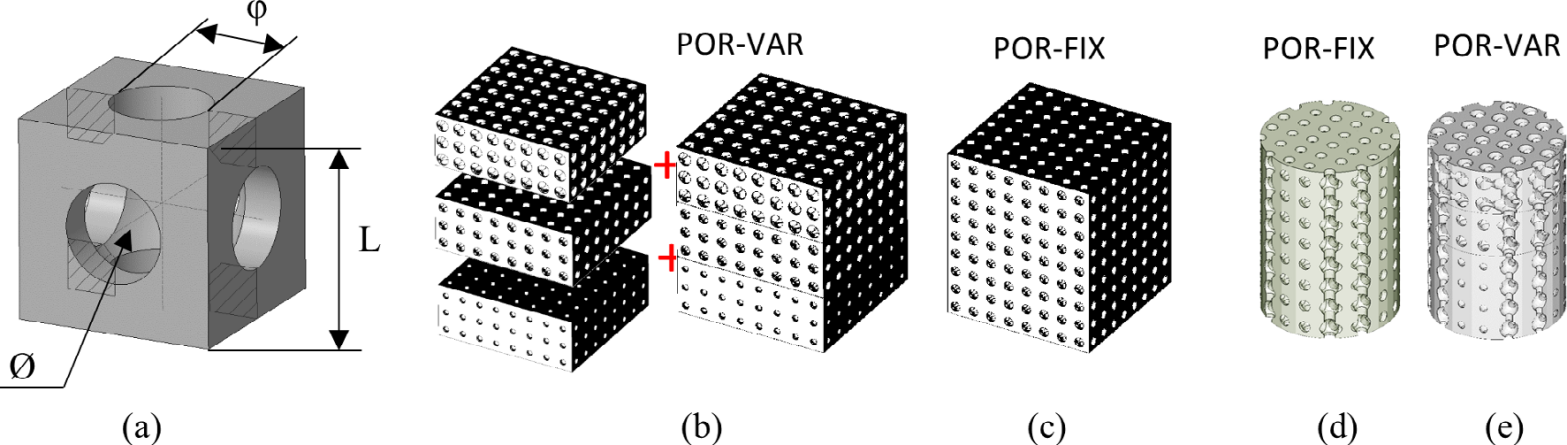
Main unit (a) and lattice structures used for compression test (b,c) and biological analysis (d,e)

The spherical hollow cubic unit type used to design the lattice scaffolds was chosen following a careful mechanical and biological analysis performed in a previous study.^38^ This unit cell (Figure 1a) is univocally characterized by three parameters: the edge of the cube (L = 1.5 mm); the diameter (Ø = 1.2 mm) of the internal spherical cavity, and the diameter (φ) of the six holes on the faces connecting the internal spherical cavity with the outside. Scaffold overall density and stiffness properties were varied by changing φ, while maintaining a fixed Ø of 1.2 mm in diameter.

Two 12 × 12 × 15 mm lattice scaffolds were designed for mechanical characterization: an uniform lattice structure (POR‐FIX) with holes diameter of 750 μm and a graded structure (POR‐VAR) obtained by stacking up three 12 × 12 × 5 mm uniform porosity layers with varying holes diameter of 500 μm, 750 μm and 1,000 μm, respectively (Figure 1b,c).

The same uniform ad graded layers configuration was used to conduct the biological tests, but the samples were cylindrical with a diameter of 9 mm and height of 12 mm (Figure 1d) to allow a perfect housing in the 48‐wells culture plates.

All samples were obtained via Selective Laser Melting (SLM MYSINT100, SISMA S.p.a., Vicenza, Italy) of atomized CoCr powder (Praxair S.T. Technology, Inc., IND) with spherical grains and chemical composition reported in the previous paper.^38^ The system is equipped with a fiber laser with a maximum power of 175 W and spot diameter of 55 μm. Production took place in a nitrogen environment with a residual oxygen content of 0.1% to minimize oxidation, using the optimized parameters of 130 W and 1,200 mm/s scanning speed for the best resolution.

Before cell seeding, the samples for biological tests were carefully washed several times with distilled water and maintained overnight under stirring, immersed in water, to facilitate the release of un‐melted powders or debris. The scaffolds were then dried and sterilized by autoclave (Getinge Disinfectation AB‐HS33 1P, Getinge Group, Roma, Italy) and pre‐wetted with osteoblast growth medium for 1 day at 37°C.

### 2.2 Mechanical properties

Global compressive properties of the two scaffolds were assessed via standards provided by American Society for Testing and Materials (ASTM E9‐09), by means of a hydraulic testing machine (Italsigma, Forli, Italy) equipped with a 100 kN load cell. The cross‐head separation rate was kept constant at 0.17 mm/min throughout the tests with a strain rate of 2·10^−4^ s^−1^ and all tests were stopped in case of sample's failure. While compressive tests return global properties of lattice structure, Digital Image Correlation technique (DIC) was used to characterize the local mechanical properties. DIC allows to estimate the local deformation of a structure by comparing images of an established surface subjected to increasing load.^22^, ^39^ Structure deformation were visually assessed by the subtle changes in the distances between recognizable features of the investigated surface. A random pattern of speckles was artificially created by spraying one lateral surface of the samples, used for the DIC analysis, with black paint on a white background. In the present study, frame sequence was captured at regular load steps (2.5–5 kN) until sample's failure. Images were taken using a digital camera (Basler, 6Mpx) and a LED array was used to ensure appropriate lighting conditions of the samples. Analysis of the images was performed with the Matlab image processing toolbox along with an open‐source subset‐based 2D DIC package (Ncorr, Matlab ver. R2019b, Mathworks). Since scaffolds showed different displacements according to the distance from the loading surface, DIC data are here presented as displacement maps in direct connection with the global properties, and as deformation maps. The latter are free from the effects of rigid displacements and effectively describe the local strain, up to the fracture of the structure.

Surface roughness of all metal samples was measured with a stylus profilometer (Alpa RT‐25; tip radius = 5 μm).

All tests were performed considering lattice samples in as‐built conditions, without post‐processing treatments, either mechanical or thermal.

### 2.3 Cell culture conditions

Normal human osteoblast cells (NHOst; LONZA, Verviers, Belgium) were maintained in osteoblast basal medium (OBM™ Osteoblast Growth and Differentiation Basal Medium; LONZA) completed with the appropriate supplements (OGM™ Osteoblasts Growth SingleQuots™ kit, LONZA), 10% fetal bovine serum (FBS, EUROCLONE, Pero, Milano, Italy), 100 U/ml penicillin, 100 μg/ml streptomycin, (SIGMA, St. Louis, MO) in standard conditions (37°C, 5%CO_2_/95%air, humidified atmosphere).

To assess biocompatibility, each porous sample was placed in 48‐well plates to avoid cells' dispersion (as previously described),^38^ statically seeded with 5 × 10^4^ cells suspended in 1 ml of medium, moved to a new 24‐well plate after 1 day and maintained in culture until 14 days.

NHOst were also seeded directly in tissue‐culture polystyrene wells as bidimensional standardized control (CTR). Medium was refreshed twice a week.

### 2.4 Cell viability and proliferation

Cell viability was observed at 1 day, 7 day, and 14 day by Alamar blue assay (Serotec, Oxford, UK) as previously reported.^38^ Samples immersed in culture medium, but without cells, were used as control for the background fluorescence.

In order to evaluate the proliferation, cells were washed with phosphatase buffer solution (PBS), detached by repeated pipetting with trypsin/EDTA (Sigma–Aldrich, UK), harvested in complete medium to stop the trypsin action, and counted in Neubauer chamber using the erythrosin vital dye, which stains the dead cells.

### 2.5 Cell morphology and scaffold colonization

Cells morphology and spreading, as well as cells/scaffold interaction, were observed after 1 day (d), 7 day and 14 day of culture by a dual approach: fluorescent labelling (fluorescein isothiocyanate –FITC‐ conjugate phalloidin, Sigma–Aldrich, Steinheim, Germany) and scanning electron microscopy (SEM, Zeiss EVO HD15 Scanning Electron Microscope, Carl Zeiss S.p.A, Italia).

Briefly, for fluorescent labelling the cultures were washed with PBS, fixed with a 4% paraformaldehyde solution in PBS for 15 min at 37°C, permeabilized in 0.5% Triton X‐100 for 15 min, again washed in PBS and labelled with a FITC‐conjugate phalloidin solution 1:100 in PBS for 30 min at 37°C. The cell cytoskeleton, to which phalloidin bounds, was visualized using an inverted microscope equipped with an epifluorescence setup (Eclipse TiU, NIKON Europe BV, NITAL SpA, Milano, Italy): by the excitation/emission setting of 488/530 nm the green fluorescence was appreciated, and the cells attached to the scaffolds, as well that in CTR condition, were easily visualized.

For SEM analysis, the samples were fixed in 2.5% glutaraldehyde, in pH 7.4 phosphate buffer 0.1 M for 1 hr at room temperature and dehydrated in a graded ethanol series. Before SEM observation, samples were air dried after hexamethyldisilazane‐based treatment.

### 2.6 Gene expression

Gene expression was observed at 1 day, 7 day and 14 day of culture. Total RNA was extracted by cells seeded on the scaffolds and by CTR using the commercial RNeasy Mini Kit (Purelink™ RNA miniKit, Ambion by Life Technologies, Carlsbad, CA), quantified by a NANODROP spectrophotometer (NANODROP 2720, Thermal Cycler, Applied Biosystem) and reverse transcribed using the Superscript Vilo cDNA synthesis kit (Life Technologies). In 10 ng of cDNA were tested in duplicate for each sample.

Gene expression was evaluated by semiquantitative PCR analysis, using the SYBR green PCR kit (QIAGEN GmbH, Hilden, Germany) in a Light Cycler 2.0 Instrument (Roche Diagnostics, GmbH, Manheim, Germany). The protocol included a denaturation cycle at 95°C for 15 min, 25 to 40 cycles of amplification and a melting curve analysis to check for amplicon specificity. The following primer sets were used: GAPDH (forward: 5′‐TGGTATCGTGGAAGGACTCA−3′, reverse: 5′‐GCAGGGATGATGTTCTGGA ‐3′), ALPL (QuantiTect Primer Assay Hs_ALPL_1_SG), TNFRSF11B (QuantiTect Primer Assay Hs_TNFRSF11B_1_SG), TNFSF11 (forward: 5′ ‐TGAGATGAGCAAAAGGCTGAG‐3′, reverse: 5′‐ AGGAGCTGTGCAAAAGGAAT‐3′), *COX2* (QuantiTect Primer Assay Hs_ PTGS2_1_SG), IL1β (QuantiTect Primer Assay Hs_IL1B_1_SG). The annealing temperature was 55°C for all the primer sets except for TNFSF11 and GAPDH (60°C and 56°C, respectively). The mean threshold cycle was determined for each sample and used for the calculation of relative expression using the Livak method (2^‐ΔΔCt^), with GAPDH as reference gene and CTR samples at 24 hr as calibrators at each experimental time.^40^

Statistical analysis was performed using R v.3.6.1 software^41^ and R packages “lme4” v. 1.1–21,^42^ “lmerTest” v.3.1^43^ “emmeans” v.1.4.1,^44^ and “ggplot2” v.3.1.1.^45^ Normal distribution (Shapiro–Wilk normality test) and homogeneity of variance (Levene test) were verified before doing data analysis. Data are presented as boxplots or Mean ± *SD* at a significant level of *p* < 0.05. Linear mixed models (LMM) were used to evaluate if there were significant interactions or effects of “material” factor (between‐subjects) and “experimental time” factor (within‐subjects, repeated measures)–on cell vitality and proliferation, and gene expression.

Pairwise comparisons of estimated marginal means (also known as least‐squares means) were carried out as post‐hoc tests to identify significant differences among Groups in term of effect size d_msw_:^46^

$$d_{\mathrm{msw}}=\frac{{\bar{Y}}_{1}-{\bar{Y}}_{2}}{\sqrt{{\mathrm{MS}}_{w}}},$$

where ${\bar{Y}}_{1}-{\bar{Y}}_{2}$ is the difference betweeen means of the considered pairwise comparison and $\sqrt{{\mathrm{MS}}_{w}}$ is the pooled *SD*. The estimator d_msw_ provides information of how many units of pooled *SD* the mean of population 1 is higher (positive value of d_msw_) or lower (negative value of d_msw_) than the population mean 2; Sidak's adjusted p‐values were calculated.

## 3 RESULTS

### 3.1 Global mechanical properties of the scaffolds

**FIGURE 2:**
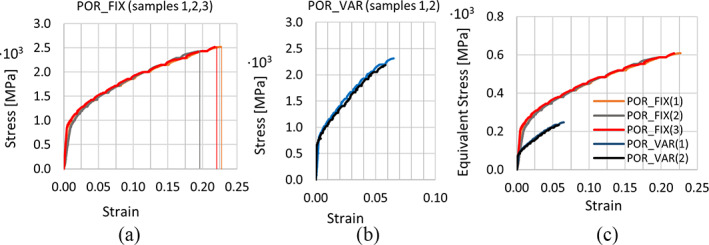
Stress–strain behaviour of POR‐FIX (a,c) and POR‐VAR (b,c) samples calculated with minimum (a,b) and equivalent (c) layer surface

For each of the two scaffolds, global mechanical properties were very consistent across samples thus supporting the repeatability of the SLM manufacturing process (Figure 2a,b; Table 1).

**TABLE 1:** Results of the compression tests

	POR_FIX (1)	POR_FIX (2)	POR_FIX (3)	POR_VAR (1)	POR_VAR (2)
Max elastic load [kN]	30			13	
Max load [kN]	87.6	84.4	87.4	35.6	33.7
Max stress [MPa]	2.52·10^3^	2.43·10^3^	2.52·10^3^	2.31·10^3^	2.19·10^3^
Equivalent US [MPa]	608	586	608	247	234
Eqivalent YS [MPa]	229	224	228	96	86
Elongation [%]	22.7	19.5	21.9	6.5	5.8

In order to compare the stress behaviour between the two scaffolds with varying cross‐sectional areas, across each layer (POR‐FIX and POR‐VAR) and across layers (POR‐VAR only), the equivalent stress was estimated by dividing the applied load over the total area (144 mm^2^) of equivalent full density structures with the mechanical properties of the two scaffolds (Figure 2c). POR‐VAR equivalent maximum stress was about 2.5 times lower than that of the POR‐FIX due to the presence of low‐density units, these probably having a large effect on the global mechanical properties (Figure 2c and Table 1). Surface roughness was consistent across samples and geometries. Ra ranged between 9–13.8 μm (mean = 10.6 ± 1.7 μm) and Rt between 47.7–62.1 μm (mean = 53.4 ± 6.0 μm).

### 3.2 Local mechanical properties

**FIGURE 3:**
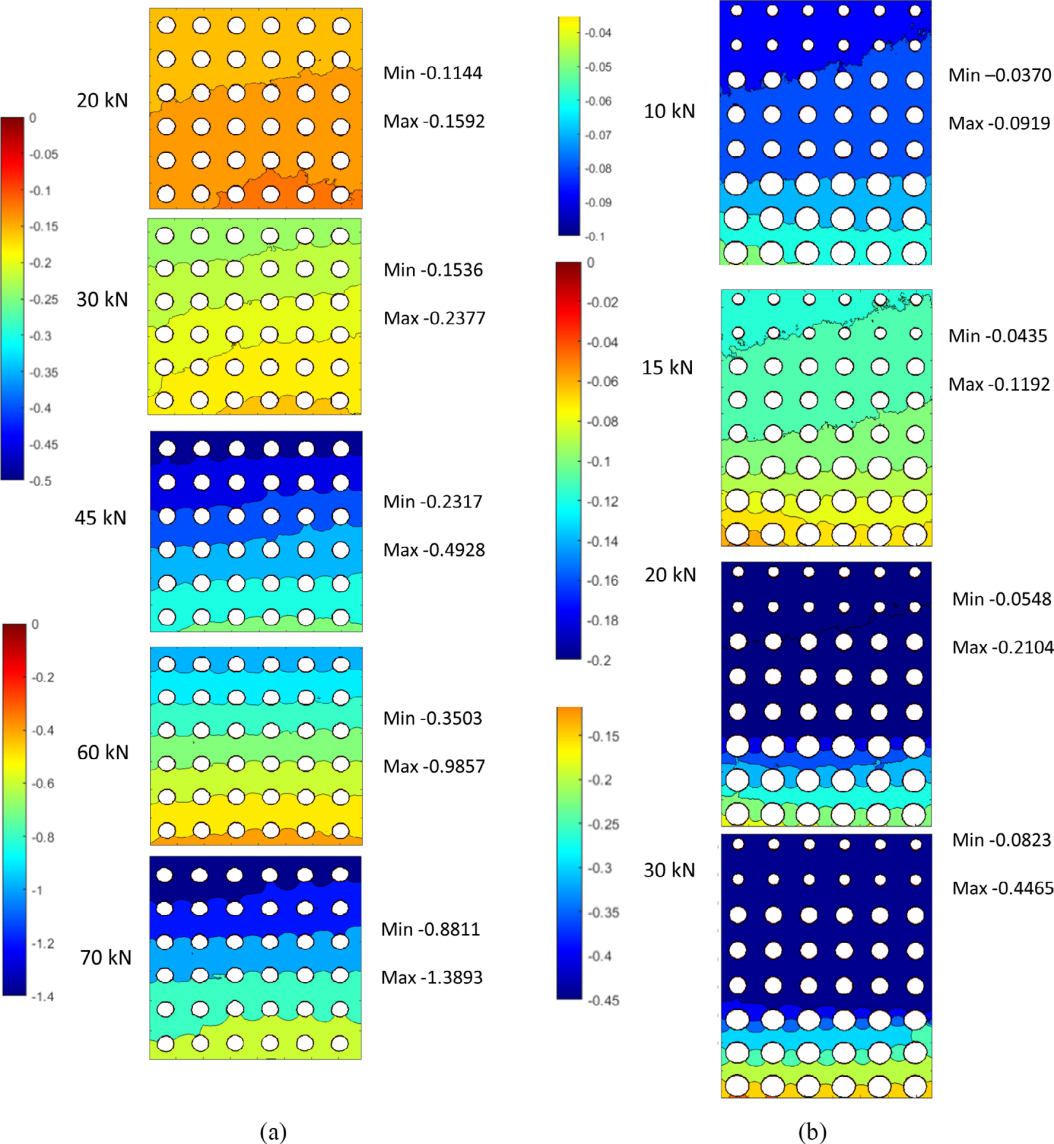
Displacement maps of POR‐FIX (a) and POR‐VAR (b) samples at different compression loads

**FIGURE 4:**
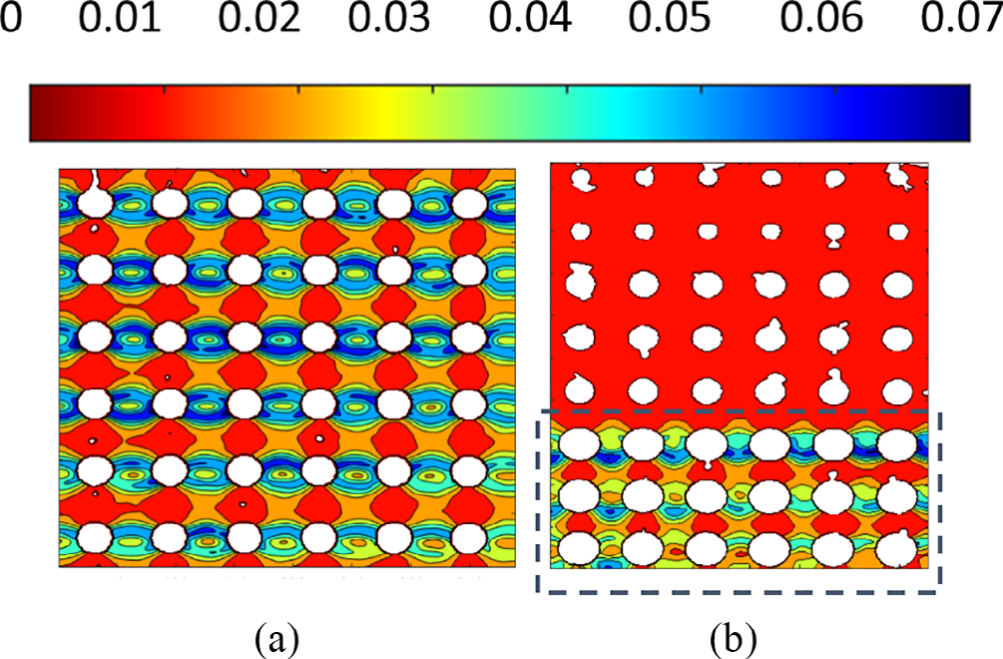
Strain maps of POR‐FIX (a) and POR‐VAR (b) structures

The DIC‐estimated local mechanical properties of the two scaffolds are reported as color maps of minimum/maximum displacement (Figure 3) of the observed surface at each loading step. An exemplary strain map is shown in Figure 4.

In both scaffolds, the maximum displacements were consistent with those from the global compressive tests. The POR‐FIX samples presented homogeneous distribution of the displacement map across layers (Figure 3a). The POR‐VAR samples presented a different behaviour in compression, with the higher density and stiffer layers moving rigidly towards the lower density layers, eventually leading to their collapse (Figure 3b).

**FIGURE 5:**
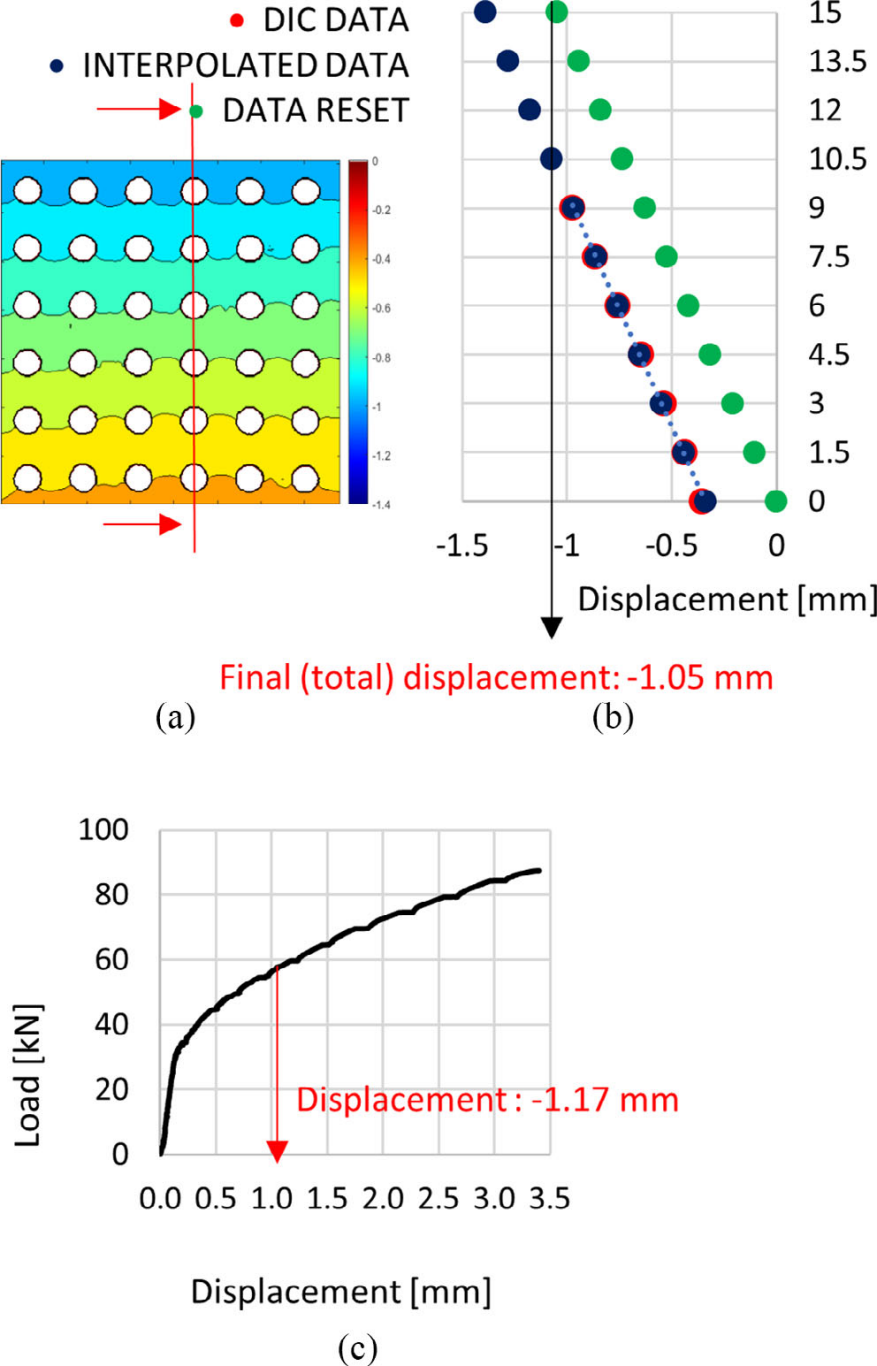
Mechanical properties obtained with the DIC method (a,b) and load/displacement curve following the compression test (c). In particular (b) shows the modified DIC data to allow comparison between local and global results. The minus sign indicates the direction of the displacement

Figure 5 allows to compare locally (Figure 5a) and globally (Figure 5c) estimated mechanical properties of the POR‐FIX scaffold. The local displacement map showed a linear behaviour along the loading direction (see Figure 5a, b). By summing up the local displacements of one section parallel to the loading direction (red line in Figure 5a), the overall compressive displacement resulted to be about 1 mm at 60kN. This displacement was subtracted from the rigid displacement of the fixed bottom surface of the scaffold (Figure 5b). DIC analysis and hydraulic machine testing demonstrated similar total displacements across all samples, for both scaffolds. In addition, the strain map (Figure 4) showed how and where the two scaffolds deformed under compressive loading. In both scaffolds, the cells were more prone to collapse in the sections parallel to the loading surface, where the stress was maximum.

### 3.3 Cell viability and proliferation

Statistical analysis showed significant interactions of selected factors on cell vitality (Alamar Blue assay: F = 1,682.70, *p* < 0.0005) and proliferation (F = 52.63, *p* < 0.0005).

**FIGURE 6:**
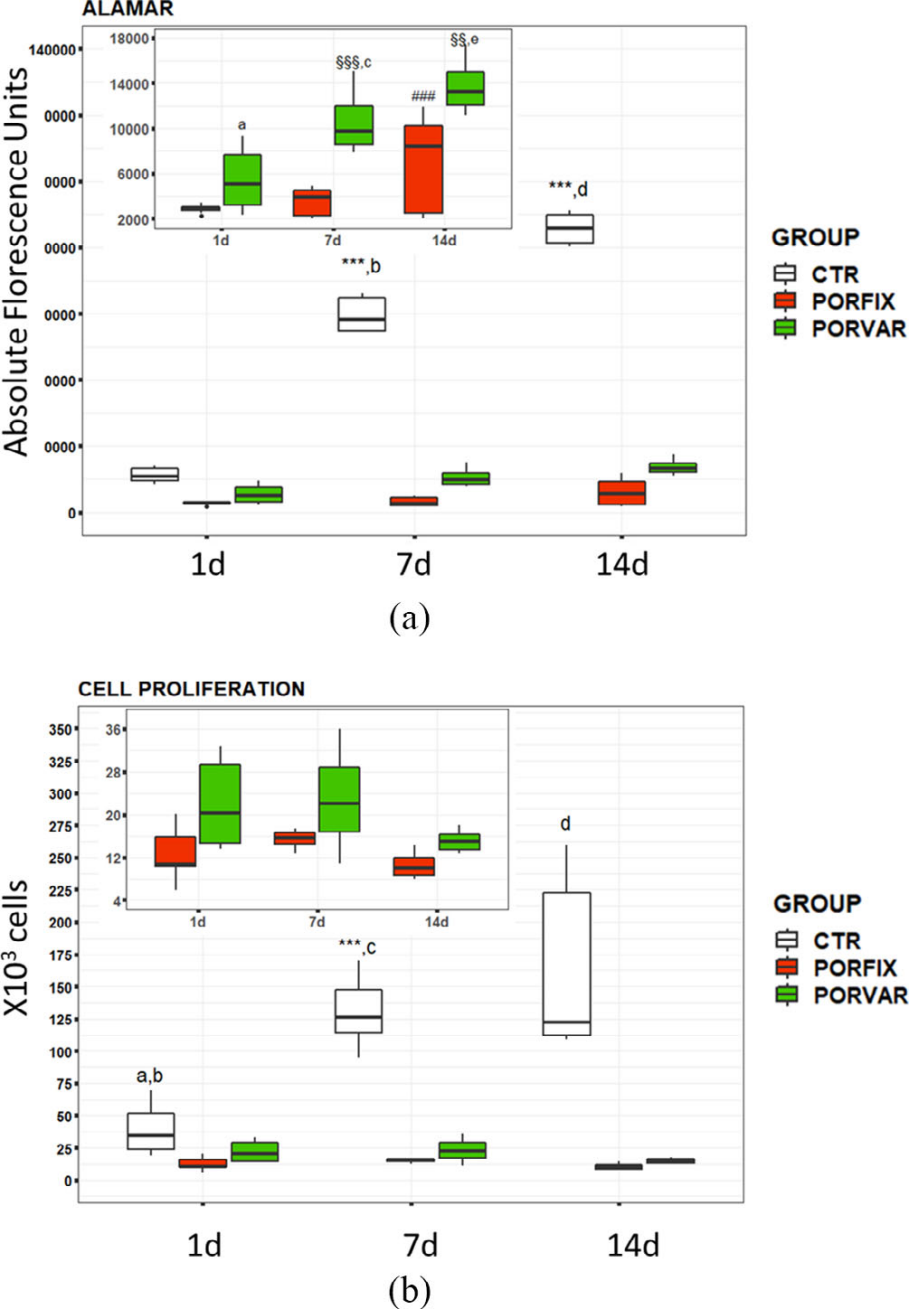
Boxplots of cell vitality (Alamar Blue) (a) and cell proliferation (b) results of NHOst cultured on POR‐FIX and POR‐VAR scaffolds compared to CTR cultures at 1, 7 and 14 days. Pairwise comparisons: Fig. 6a: ***7 day versus 1 day and 14 day versus 7 day for CTR (p < 0.0005); ###14 day versus 7 day (p < 0.0005) for POR‐FIX; §§§7 day versus 1 day (p < 0.0005) and §§14 day versus 7 day (p < 0.005) for POR‐VAR; (a), POR‐VAR versus POR‐FIX at 1 day (p < 0.0005); (b) CTR versus POR‐VAR and POR‐FIX at 7 day (p < 0.0005); (c) POR‐VAR versus POR‐FIX at 7 day (p < 0.0005); (d), CTR versus POR‐FIX and POR‐VAR at 14 day (p < 0.0005); (e) POR‐VAR versus POR‐FIX at 14 day (p < 0.0005). Fig. 6b: ***7 day versus 1 day for CTR (p < 0.0005); (a), CTR versus POR‐FIX at 1 day (p < 0.0005); (b), CTR versus POR‐VAR at 1 day (p < 0.005); (c), CTR versus POR‐FIX and POR‐VAR at 7 day (p < 0.0005); (d), CTR versus POR‐FIX and POR‐VAR at 14 day (p < 0.0005)

In particular, the cell count related to the lattice samples showed a substantial stability over time, but always higher values on POR‐VAR than POR‐FIX, although not statistically significant (Figure 6b). The increasing values over time observed in CTR group, significantly higher than those of POR‐FIX and POR‐VAR, are probably due to the wider available culture surface of the bottom well, considered the gold standard for cell culture and representing the internal control of the system.

Conversely to what observed for cell proliferation, cell activity on POR‐VAR scaffolds at each timepoint was significantly higher than cell activity on POR‐FIX. Furthermore, a regular trend of increase was observed for cells on POR‐VAR between 1 day and 14 day, and only a partial increase, between 7 day and 14 day, for POR‐FIX. (Figure 6a).

### 3.4 Cell morphology and scaffold colonization

Cell spreading and scaffold colonization were appreciated at all experimental timepoints by FITC‐ conjugate phalloidin, useful to evidence the cell cytoskeleton.

Already after 1 day both scaffolds appeared well colonized on the seeding surface, with the cells elongated in the effort to cover the available space on the top of materials. Typically, cells presenting a stretched shape are considered to be alive, whereas dead cells round up and lose adhesion from the substrate.

**FIGURE 7:**
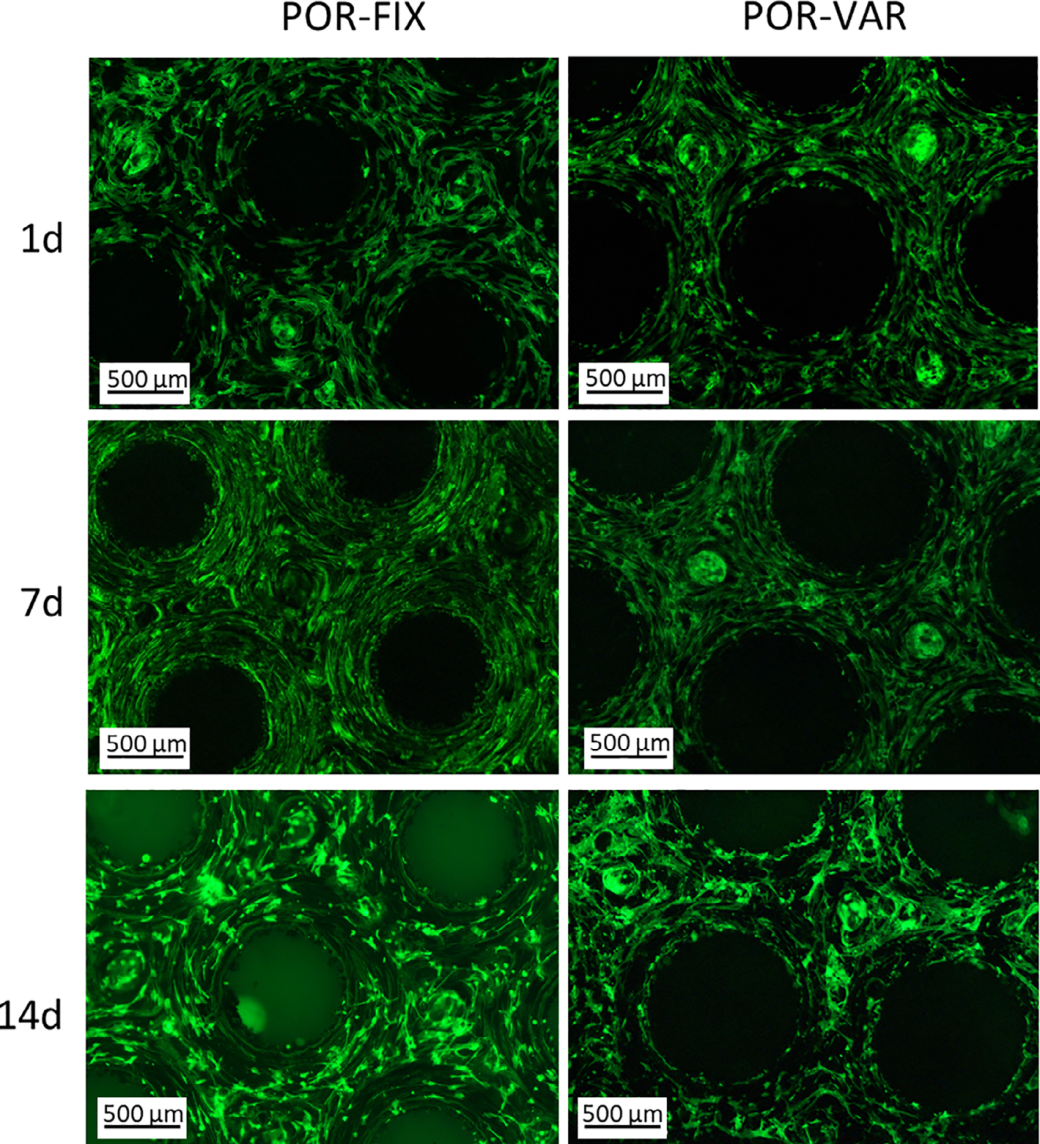
Images of NHOst labelled with fluorescein isothiocyanate (FITC), on scaffolds' surfaces (POR‐FIX on the left and POR‐VAR on the right), at 1, 7 and 14 days after seeding. Magnification 4×; scalebar: 500 μm

At 4× magnification, a mostly regular cells distribution was sometimes interrupted only by small empty areas (Figure 7). The images at 7 day and 14 day of culture confirmed what observed at earlier timepoints, with a cell density similar to that at 1 day.

**FIGURE 8:**
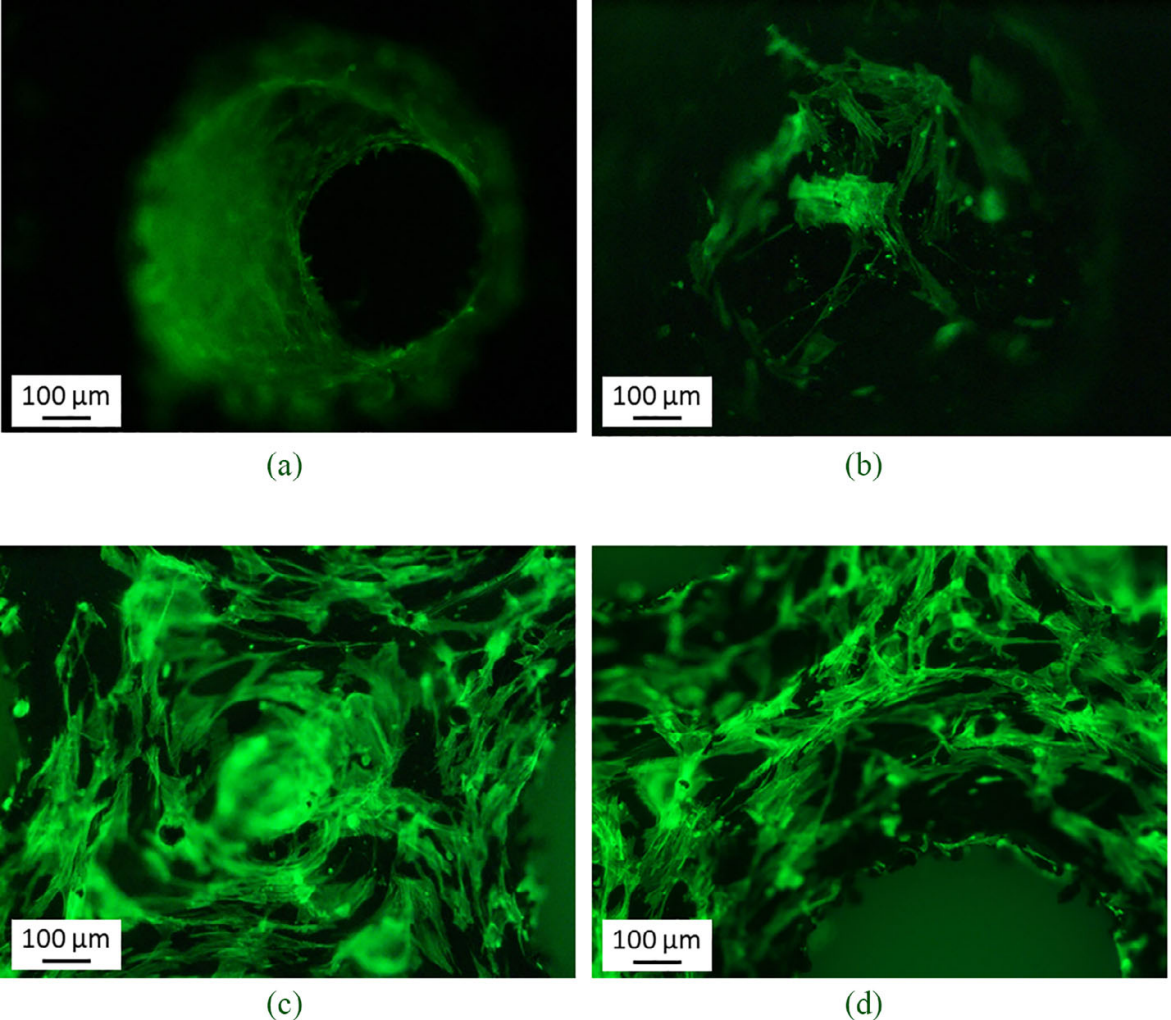
Details of cell organization on both lattice structures: NHOst forming cell layer on a pore (a) and cell–cell connection inside a pore (b); dense NHOst culture on scaffold surface (c) and contouring a pore edge (d). Magnification: 10×, scalebar: 100 μm

At higher magnification (10×) it was possible to observe the intricate osteoblasts' organization on the CoCr surface and around the pores (Figure 8), while cavities colonization could not be fully appreciated, due to instrumental limitations in the analysis of three‐dimensional components. Thus, cells adhesion can be observed only on the most superficial samples layer, with cells creating a uniform layer and trying to fill the samples pores (Figure 8).

Similarly, morphological qualitative analysis performed by SEM confirmed the observations reported above: NHOst cells colonized both POR‐FIX and POR‐VAR scaffolds (further details in the Discussion section and in Figure 11). The scaffolds appeared to provide the correct substrate and microenvironment for bone forming cells as these were visible on the top surface and inside the pores, until the last endpoint, with their typical spreading feature. In particular, the comparison between the scaffolds with different porosity revealed a slightly more pronounced cellular colonization of the deeper visible levels in the POR‐VAR.

### 3.5 Gene expression

The analysis of some key genes for the osteoblast activity allowed to better understand the reaction of these cells to the scaffolds. Significant interactions of selected factors were found on *ALPL* (F = 61.81, *p* < 0.0005), *COX2* (F = 1,415, p < 0.0005), and *IL1β* (F = 5.38, *p* < 0.05), while significant effects for *OPG/RANKL* (material: F = 6.41, *p* = 0.004; experimental time: F = 62.39, *p* < 0.0005) ratio was observed.

**FIGURE 9:**
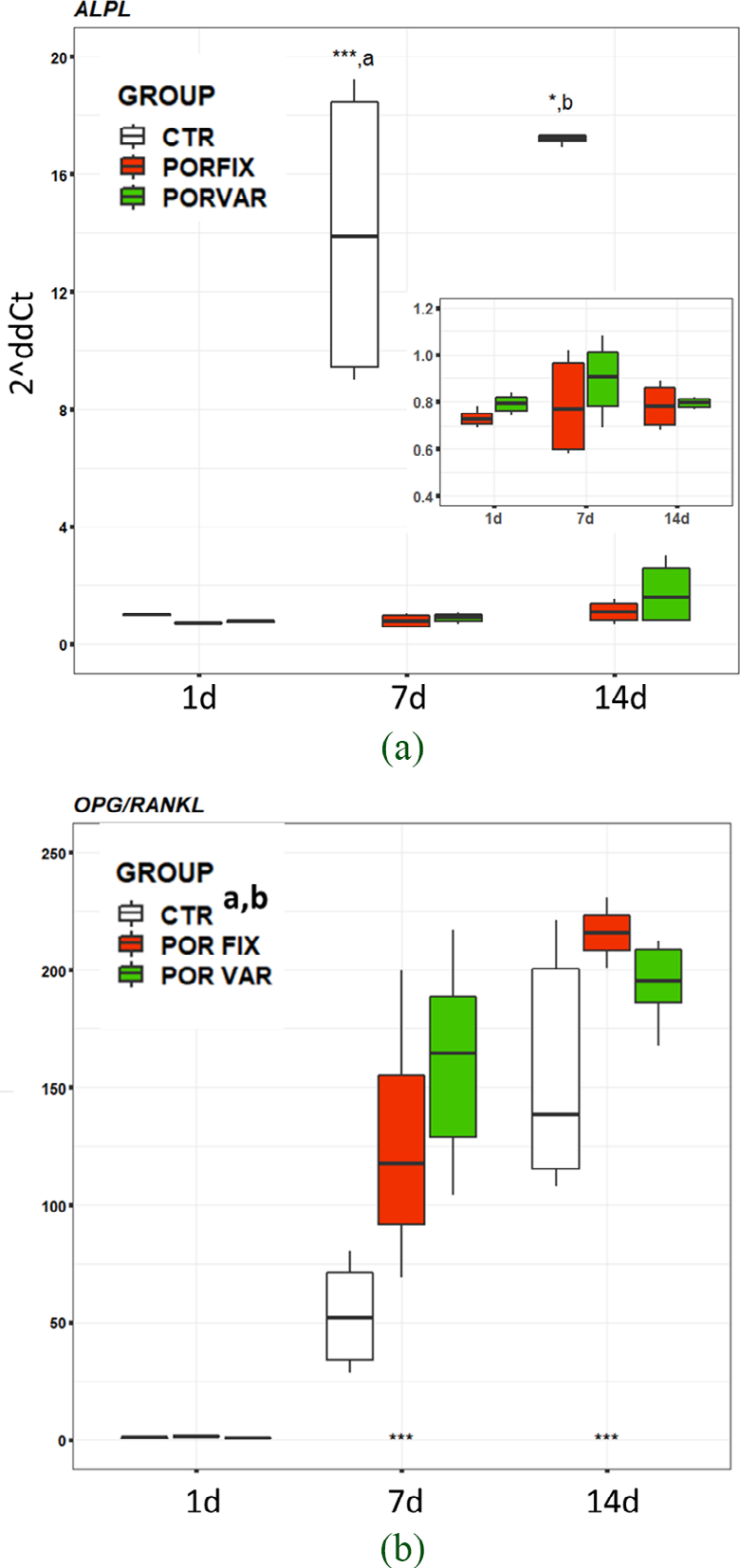
Boxplots of *ALPL* gene expression and *OPG/RANKL* ratio of NHOst cultured on POR‐FIX and POR‐VAR scaffolds compared to CTR cultures at 1, 7 and 14 days. Pairwise comparisons: Fig. 9a: ***7 day versus 1 day (p < 0.0005); *14 day versus 7 day for CTR (p < 0.05); (a) CTR versus POR‐FIX and POR‐VAR at 7 day (p < 0.0005); (b) CTR versus POR‐FIX and POR‐VAR at 14 day (p < 0.0005). Fig. 9b: ***7 day versus 1 day and 14 day versus 7 day (p < 0.0005), independently by scaffolds; (a), (b) POR‐FIX (p<0.05) and POR‐VAR (p < 0.005) versus CTR, independently by experimental time

ALPL expression could well describe the role of cell density for this gene. In CTR condition, at 7 day, *ALPL* expression was significantly higher than that observed on scaffolds where, consistently with the proliferation pattern, it was steady over time. At 14 day, confirming a typical *ALPL* pattern, it slightly decreased also in CTR. (Figure 9a).

Interestingly, the *OPG/RANKL* expression ratio, which shows the ratio of osteoblasts to osteoclasts activity, increased significantly over time (7 day vs 1 day: d = 1.7, *p* < 0.0005; 14 day vs 7 day: d = 2.4, *p* < 0.0005) in all groups. In particular, both POR‐FIX and POR‐VAR showed significantly higher values than CTR (d = 1.3, *p* < 0.05 and d = 1.4, *p* < 0.005, respectively), and no differences were observed between the two scaffolds (Figure 9b).

**FIGURE 10:**
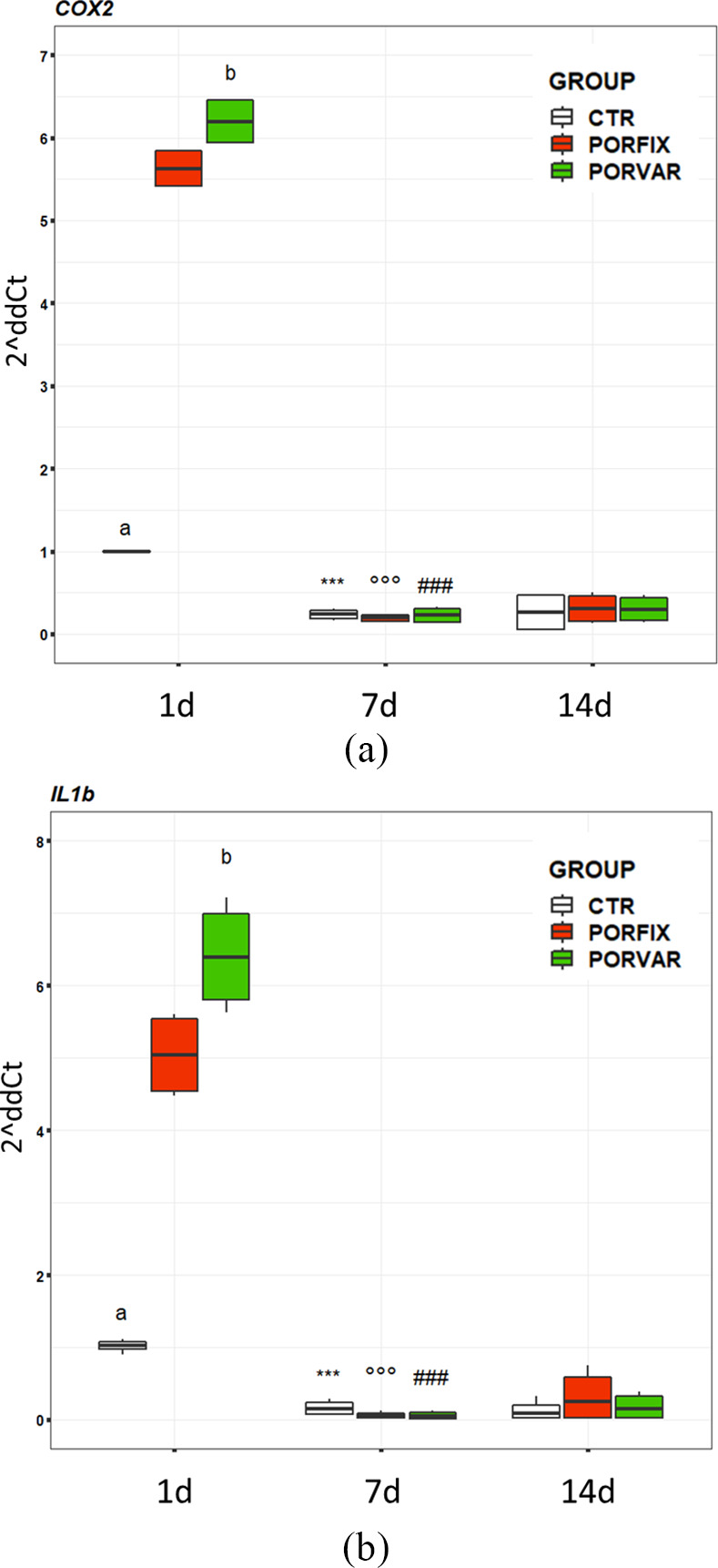
Boxplots of *COX2* and *IL1β* gene expressions of NHOst cultured on POR‐FIX and POR‐VAR scaffolds compared to CTR cultures at 1, 7 and 14 days. Pairwise comparisons. ***7 day versus 1 day for CTR (*p* < 0.0005); °°°7 day versus 1 day for POR‐FIX ( *p* < 0.0005) and ^###^7 day versus 1 day for POR‐VAR (*p* < 0.0005). (a), CTR versus POR‐FIX and POR‐VAR at 1 day (*p* < 0.0005); (b) POR‐VAR versus POR‐FIX at 1 day (*p* < 0.0005)

*COX2* (Figure 10a) and *IL1β* (Figure 10b) analysis provided preliminary albeit useful information on the osteoblast reaction to the novel scaffolds, highlighting the inflammatory response that could be triggered in “nonstandard” culture conditions. This response was very similar across all groups for both inflammatory markers.

More in detail, at 1 day, the osteoblasts responded with a *COX2* and *IL1β* expression significantly higher in POR‐FIX and POR‐VAR than in CTR, with POR‐VAR showing significantly higher values than POR‐FIX (Figure 10). At 7 day however, the expression of both genes was significantly decreased in all groups (*COX2*: d = −7.2–−55.1, p < 0.0005; *IL1β*: d = −3.2–−24.4, *p* < 0.0005). These expression values were maintained also at 14 day, when no difference was detected between scaffolds and CTR.

**FIGURE 11:**
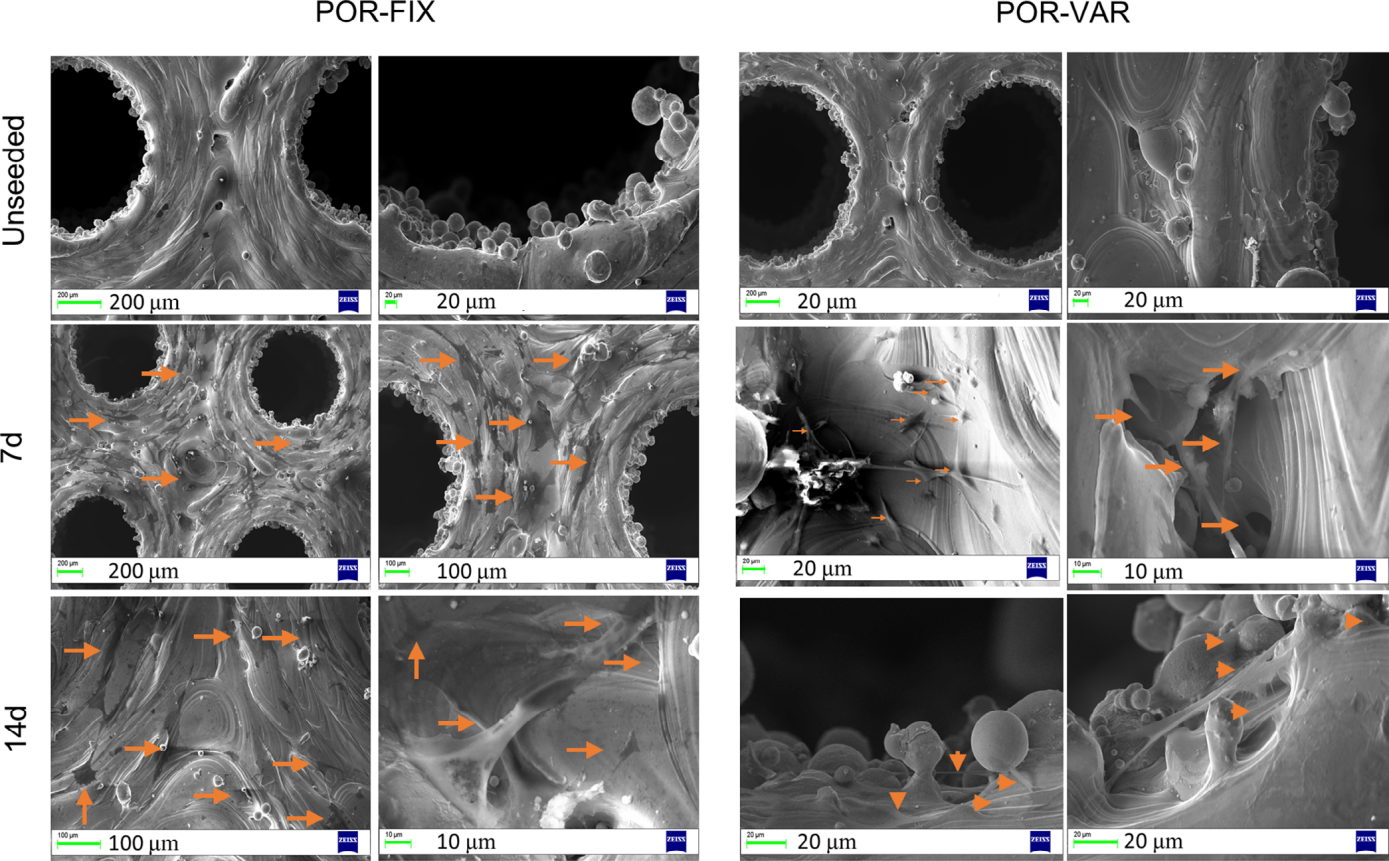
SEM micrographs of the samples showing the structure (unseeded scaffold) and colonization grade of CoCr POR‐FIX scaffold seeded with NHOst (arrows) at 7 day and 14 day. Scale bars are reported on the low left side of each image

## 4 DISCUSSION

While the mechanical and biological properties of Ti alloys lattices have been widely investigated with respect to cell types and porosity,^47^ little is still known on the biocompatibility and mechanical suitability of CoCr porous scaffolds obtained via SLM for orthopedic implants. In a previous investigation by the present authors,^38^ optimization of the SLM manufacturing parameters resulted in 60%–70% CoCr lattice with sufficient accuracy with respect to the nominal design. Since no significant differences were previously found in proliferation and viability of osteoblast‐like cells (Saos2) between trabecular scaffolds and those based on geometrical unit cells, the spherical hollow cubic cell was used in the present investigation because of its simple parameterization and higher reproducibility. Compared to the human cortical and cancellous bone, CoCr is a stiff material characterized by a Young modulus of about 200GPa which is twice as large as that of Ti alloys. This intrinsic material property affects the stress and strain distribution between implant and bone, therefore implants must be optimally designed to reduce the overall stiffness without compromising strength and durability. At a same time, these designs must guarantee a suitable environment at the implant‐bone interface for osteoblast colonization and proliferation. The results of the present work should be interpreted in the context of a larger multi‐disciplinary investigation aimed at improving current understanding on CoCr lattice having the sufficient strength and minimizing the stress shielding of orthopedic implants, whilst ensuring suitable biocompatibility properties.

According to the Maxwell constant M,^48^ the mechanical behavior of lattice structures in compression can be classified as bending‐dominated (M < 0) or stretch‐dominated (M > 0). Although the unit element used here has 12 struts and 8 nodes and thus presents a negative Maxwell constant, the stress–strain curve reported in Figure 2 shows a stretch‐dominated trend for both POR‐FIX and POR‐VAR structures. This is probably due to the single strut direction with respect to the load vector: the presence of vertical Z‐struts aligned with the compressive loading direction makes the structure more subjected to a stretch‐dominated mechanism.^49^ The Maxwell criteria does not appear to be sufficiently reliable to predict the real mechanical behavior of lattice, in particular in case of nonconventional unit cell and graded porosity.^49^ While POR‐FIX and POR‐VAR structures presented a similar global mechanical behavior, maximum stress and deformation were significantly different. Standard compression tests, however, do not allow to extract more detailed information on properties of such variable‐geometry structures, other than the global stress–strain pattern. DIC analysis, however, allowed to compare uniform and graded lattice structures in terms of local deformation behavior, which cannot be directly inferred from standard compressive testing. The lower stress and strain observed in POR‐VAR samples were due to the mechanical properties of the 1,000 μm pore layers. While presenting the same average porosity, thus allowing the same potential osseointegration capability of the uniform porosity scaffolds, POR‐VAR scaffolds appeared to be more deformable than POR‐FIX scaffolds and, unlike what observed in the latter, deformation varied across layers with different porosity. Varying the local stiffness behaviour could be an effective mechanism to reduce the stress shielding of orthopedic implants.

In terms of biological response in vitro, the assays performed here should be assessed also in light of the outcome and in continuity with the previous study.^38^ Although affected by the intrinsic limitations of in vitro models, the use of primary osteoblast cells should be more representative of implants biocompatibility in the real clinical scenario. These are primarily fixated to the cortical bone but, according to the specific surgical treatment, often extend into lattice cancellous bone.

Active cell proliferation and typical osteoblast morphology observed in the control group (intended as internal control) at all‐time points confirmed the quality and suitability of the chosen cellular model. The wide and regular surface of the well bottom provided ideal culture conditions, with respect to the smaller and irregular surface available on the lattice samples, thus explaining the reason for the highest values observed in CTR group for proliferation and activity. Nevertheless, fluorescent imaging revealed complete covering of the samples upper surface in both scaffolds, suggesting that the surface macro‐topography appears to be adequate for cells colonization. A preliminary evaluation aimed at normalizing cell viability and proliferation with respect the colonized surface, revealed better results for the POR‐VAR scaffolds (data not shown). This analysis will be further explored in future investigations.

Similarly, SEM images clearly confirmed how NHOst colonized the samples surfaces and the first lattice layer of both scaffolds at each time point, but slightly more in the POR‐VAR. The images were consistent with what reported for cell proliferation, and even more for cell activity (Figure 11).

Although the full covering of the statically‐seeded samples' surface by osteoblasts could hamper the exchange of oxygen and nutrient supplies and the colonization of deeper layers, in light of the in vitro model limitations, this has been considered a desirable outcome improving scaffolds colonization and osseointegration.^50^ Figure 7 effectively shows how this phenomenon was observed in the present structures, albeit only in some samples.

Osteoblasts activity was evaluated by the expression of key genes, such as *ALPL, OPG*, and *RANKL*. In particular, *ALPL* expression appeared to be consistent, at least partially, with what observed for cells proliferation: while in the control group *ALPL* expression significantly increased after 7 day, thus indicating a typical osteoblast activity, the cells on both scaffolds maintained the same low‐gene expression level. This could be explained by the high cell density of the control condition, not yet reproducible at these timepoints on the narrow and rough surfaces of the scaffolds. In addition, it has been observed that osteoblasts, without other cellular types necessary to complete the bone microenvironment and in absence of chemically‐functionalized surfaces, are poorly stimulated to express *ALP*.^51^, ^52^

The present study also investigated how SLM produced CoCr scaffolds could elicit inflammatory reaction in osteoblasts and could affect the complex balance involving bone formation and resorption. Regarding the fine crosstalk between osteoblasts and osteoclasts, it is widely known the crucial role of the *RANKL/RANK/OPG* system: nuclear factor‐kappa B (NF‐κB) ligand (*RANKL*) has a fundamental role because able to bind *RANK* receptor on preosteoclast membrane, so triggering osteoclast maturation, or osteoprotegerin (*OPG*): a decoy receptor that limits the biologic activity of *RANKL*, competing with it.^53^ In the present study, the ratio of *OPG/RANKL* expression significantly increased in the control over time, but also in both scaffolds. Furthermore, the larger *OPG/RANKL* ratio observed in the CoCr lattice samples with respect to control suggest that these structures are effective in promoting osteosynthesis. Aseptic mobilization, one of the main issues contributing to failure of endoprostheses, depends also on the fine biological system regulating osteosynthesis and osteolysis,^54^, ^55^ which in turn could be affected by a possible inflammatory response induced by free nanoparticles or material debris.^56^ SLM technique, in fact, produces irregular surfaces due to resolution allowed by the laser spot diameter with respect to the powder size. Although the main cellular inflammatory response can be generated by the presence of few μm sized debris,^57^ the possible presence of unmelted CoCr powder could warrant further evaluation. Therefore, the expression of *IL1β* and *COX2* could indicate an osteoblast‐mediated inflammatory response, triggered by the unmelted powder particles with micrometric and sub‐micrometric diameter (less than 300 nm) present in as‐built SLM components. These free particles could stimulate osteolysis^55^ and should be further investigated with respect to the manufacturing technique. Indeed, 1 day after scaffolds seeding, osteoblasts showed a clear activation of *IL1β* and *COX2* expression, which was not observed in the control. At 7 day and 14 day, however, no significant difference in the expression of these genes was observed between control and porous samples, thus suggesting the capability of osteoblasts to acclimatise to the CoCr environment and restore a normal noninflammatory response.

## 5 CONCLUSIONS

Following the compression tests, the CoCr graded lattice structure presented an equivalent maximum stress about 2.5 times lower than that in the uniform structure and appeared more deformable, with a stratified strain behaviour associated to its porosity and to the unit cell geometry. The stiffness of the entire structure or of specific regions can be optimized according to the application.

Both uniform and graded structures provide the osteoblasts with an environment suitable for adhesion and proliferation, capable to support a favorable *OPG/RANKL* ratio and a self‐limiting gene expression of the analyzed inflammatory mediators *IL1β* and *COX2*.

Both lattice structures presented good biocompatibility properties, but graded structures seem to offer a better solution to improve the stress distribution between CoCr orthopedic implants and bone.

## Data Availability

The data that support the findings of this study are available from the corresponding author upon reasonable request.
